# The omega-3 postbiotic *trans*-10-*cis*-15-octadecadienoic acid attenuates contact hypersensitivity in mice through downregulation of vascular endothelial growth factor A

**DOI:** 10.3389/fcimb.2024.1355679

**Published:** 2024-05-22

**Authors:** Azusa Saika, Takahiro Nagatake, Shigenobu Kishino, Nahoko Kitamura, Tetsuya Honda, Koji Hosomi, Prabha Tiwari, Eri Node, Soichiro Kawai, Saki Kondo, Kei Ishida, Kenji Kabashima, Jun Ogawa, Jun Kunisawa

**Affiliations:** ^1^ Laboratory of Vaccine Materials and Laboratory of Gut Environmental System, Microbial Research Center for Health and Medicine, National Institutes of Biomedical Innovation, Health and Nutrition (NIBIOHN), Ibaraki, Japan; ^2^ Institute of Molecular and Cell Biology, Agency for Science, Technology and Research, Singapore, Singapore; ^3^ Laboratory of Functional Anatomy, Department of Life Sciences, School of Agriculture, Meiji University, Kawasaki, Japan; ^4^ Division of Applied Life Sciences, Graduate School of Agriculture, Kyoto University, Kyoto, Japan; ^5^ Department of Dermatology, Hamamatsu University School of Medicine, Hamamatsu, Japan; ^6^ Department of Microbiology and Immunology, Keio University School of Medicine, Tokyo, Japan; ^7^ Graduate School of Pharmaceutical Sciences, Osaka University, Suita, Japan; ^8^ Department of Dermatology, Graduate School of Medicine, Kyoto University, Kyoto, Japan; ^9^ International Vaccine Design Center, The Institute of Medical Science, The University of Tokyo, Tokyo, Japan; ^10^ Graduate School of Medicine, Graduate School of Dentistry, Graduate School of Science, Osaka University, Suita, Japan; ^11^ Department of Microbiology and Immunology, Graduate School of Medicine, Kobe University, Kobe, Japan; ^12^ Research Organization for Nano and Life Innovation, Waseda University, Shinjuku, Tokyo, Japan; ^13^ Graduate School of Biomedical and Health Sciences, Hiroshima University, Higashi-Hiroshima, Japan

**Keywords:** omega-3 fatty acid, intestinal bacteria, postbiotics, contact hypersensitivity, vascular endothelial growth factor

## Abstract

Intestinal bacteria metabolize dietary substances to produce bioactive postbiotics, among which some are recognized for their role in promoting host health. We here explored the postbiotic potential of two omega-3 α-linolenic acid–derived metabolites: *trans*-10-*cis*-15-octadecadienoic acid (t10,c15-18:2) and *cis*-9-*cis*-15-octadecadienoic acid (c9,c15-18:2). Dietary intake of lipids rich in omega-3 α-linolenic acid elevated levels of t10,c15-18:2 and c9,c15-18:2 in the serum and feces of mice, an effect dependent on the presence of intestinal bacteria. Notably, t10,c15-18:2 mitigated skin inflammation in mice that became hypersensitive after exposure to 2,4-dinitrofluorobenzene, an experimental model for allergic contact dermatitis. In particular, t10,c15-18:2—but not c9,c15-18:2—attenuated ear swelling and edema, characteristic symptoms of contact hypersensitivity. The anti-inflammatory effects of t10,c15-18:2 were due to its ability to suppress the release of vascular endothelial growth factor A from keratinocytes, thereby mitigating the enhanced vascular permeability induced by hapten stimulation. Our study identified retinoid X receptor as a functional receptor that mediates the downregulation of skin inflammation upon treatment with t10,c15-18:2. Our results suggest that t10,c15-18:2 holds promise as an omega-3 fatty acid–derived postbiotic with potential therapeutic implications for alleviating the skin edema seen in allergic contact dermatitis–induced inflammation.

## Introduction

**Figure 1:**
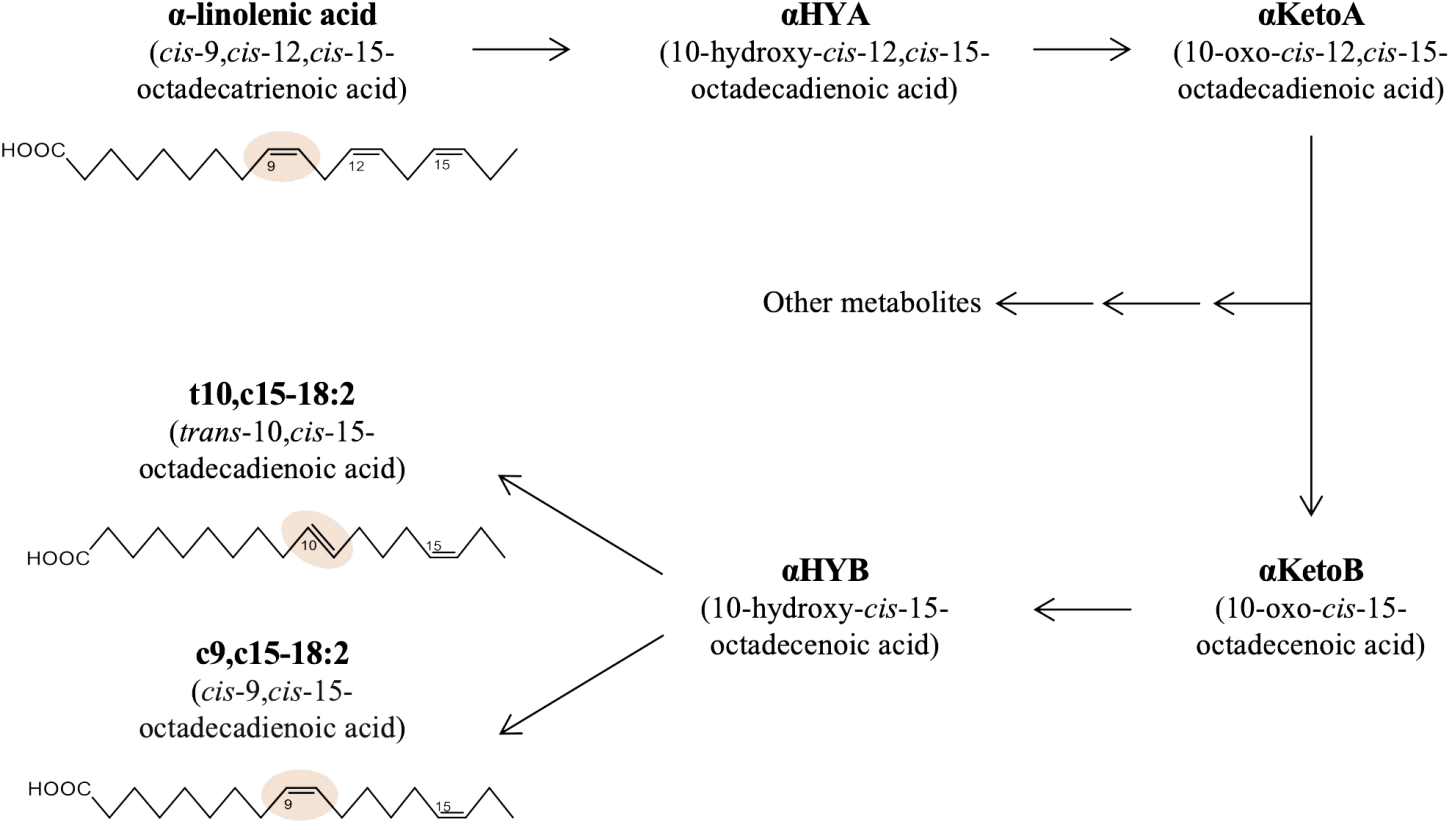
Production pathway of t10,c15-18:2 and c9,c15-18:2 from α-linolenic acid in bacteria. The metabolic pathway and structures of the fatty acids central to this research, namely α-linolenic acid, t10,c15-18:2, and c9,c15-18:2.

A growing body of evidence reveals the profound influence of intestinal bacteria on host health and diseases (Afzaal et al., 2022). Even though intestinal bacteria dwell primarily in the intestinal lumen and do not infiltrate systemically, they markedly influence host health beyond the intestine (Agus et al., 2021). Recent studies suggest that the bioactive metabolites of dietary materials converted by intestinal bacteria, termed ‘postbiotics,’ have systemic effects in the host (Aguilar-Toalá et al., 2018; Peluzio et al., 2021). We recently found that 10-oxo-*cis*-12-*cis*-15-octadecadienoic acid (αKetoA), an intermediate metabolite of α-linolenic acid via saturation metabolism by intestinal bacteria, exerts potent anti-inflammatory activities on macrophages and suppresses the pathogenesis of contact hypersensitivity and diabetes (Nagatake et al., 2022). αKetoA can be further metabolized by *Lactobacillus plantarum* to yield *trans*-10-*cis*-15-octadecadienoic acid (t10,c15-18:2) and *cis*-9-*cis*-15-octadecadienoic acid (c9,c15-18:2) as final products due to saturation metabolism of α-linolenic acid in multiple steps, as shown in **Figure 1** (Kishino et al., 2013; Tsuji et al., 2022). However, the bioactivities of these metabolites have not been investigated.

Allergic contact dermatitis, a prevalent inflammatory skin disease with a lifetime prevalence of up to 20% (Weidinger et al., 2018; Chamani et al., 2023), is recognized as a significant occupational skin disease, necessitating the development of preventive and therapeutic strategies. In the context of allergic contact dermatitis, allergenic components known as haptens sensitize the skin immune system upon initial contact. Subsequent exposures provoke T cell–mediated immune reactions. Clinically, allergic contact dermatitis manifests as swelling, or ‘spongiosis,’ and irritation, with an increase in vascular permeability.

In this study, we sought to elucidate the beneficial effects of t10,c15-18:2 and c9,c15-18:2 as postbiotics with the potential to regulate host immune responses during inflammation. We investigated whether their production depends on the presence of intestinal microbiota after dietary intake of omega-3 α-linolenic acid. Using a mouse model of 2,4-dinitrofluorobenzene (DNFB)-induced contact hypersensitivity, we further explored the physiologic roles of these metabolites in the context of allergic contact dermatitis.

## Materials and methods

### Animals

For lipidomics analysis, we purchased male germ-free (GF) mice and control ICR mice (age, 6 weeks) from Japan SLC (Hamamatsu, Japan); these mice were maintained for 2 months on chemically defined diets containing 4% (wt/wt) dietary oil comprising soybean oil or linseed oil (Oriental Yeast, Tokyo, Japan). GF mice were housed under GF conditions at Oriental Bioservice (Kyoto, Japan), and control mice were housed under specific-pathogen-free (SPF) conditions at the NIBIOHN (Osaka, Japan).

For the contact hypersensitivity model, we obtained female wild-type C57BL/6 mice (age, 6–8 weeks) from SLC (Shizuoka, Japan) and housed them in an SPF animal facility at NIBIOHN for at least 1 week before their use in experiments. In this study, female mice were chosen for the contact hypersensitivity model due to their lower aggression levels compared to males (Schwarz et al., 2023). These mice had ad libitum access to distilled water and a commercially available standard diet (FR2, Funabashi Farm, Chiba, Japan) under conditions of 22–24°C, 50%–60% humidity, and a 16:8-h light:dark cycle. Mice were euthanized by cervical dislocation under anesthesia with isoflurane (AbbVie Inc., North Chicago, Illinois, USA). All experiments were performed in accordance with the guidelines of the Animal Care and Use Committee and the Committee on the Ethics of Animal Experiments at NIBIOHN.

### Murine contact hypersensitivity model

The model was generated as described previously (Nagatake et al., 2018; Saika et al., 2021). Briefly, on day 0 the shaved abdominal skin of C57BL/6 mice was treated with 25 μL of 0.5% (vol/vol) DNFB (Nacalai Tesque, Kyoto, Japan) in 4:1 acetone:olive oil (Nacalai Tesque). On day 5, both sides of the ears were challenged with 10 μL of 0.2% (vol/vol) DNFB. On day 7, ear thickness was measured with a micrometer (MDC-25MJ 293-230, Mitsutoyo, Kawasaki, Japan). To evaluate fatty acid activity, the ear skin of mice was treated topically with t10,c15-18:2, or c9,c15-18:2 (both produced from α-linoleic acid by using microbial enzymes) (Kishino et al., 2003; Tsuji et al., 2022); these compounds were dissolved in 50% (vol/vol) ethanol in phosphate-buffered saline (PBS) and provided at a dose of 1 µg/animal at 30 min before sensitization with DNFB on day 0 and before elicitation with DNFB on day 5. To assess fatty acid activity after the challenge on day 5, t10,c15-18:2 was topically applied to the ear skin on day 6. Control mice received 50% (vol/vol) ethanol in PBS as a vehicle control. In another experiment, we topically administered the retinoid X receptor (RXR) pan-antagonist HX531 (Cayman Chemical) at a dose of 40 nmol to the ear skin of mice. The HX531 was prepared in a solution containing 50% (vol/vol) dimethyl sulfoxide and 25% (vol/vol) ethanol in PBS. This application occurred 60 min before the fatty acid treatment, with 15 μL of the solution applied to both sides of the ears. Ear swelling was calculated as: (ear thickness [mm] after DNFB application on day 7) – (ear thickness [mm] before DNFB application on day 0).

### Cell isolation and flow cytometric analysis

Cells were isolated from ear tissue and their flow cytometric analysis was performed as described previously (Saika et al., 2021). Ears were split into dorsal and ventral skin, cut into small pieces by using scissors, and incubated in 2 mg/mL collagenase (Wako Pure Chemicals, Osaka, Japan) in RPMI 1640 medium containing 2% (vol/vol) newborn calf serum (Equitech Bio, Kerrville, Texas, USA) for 90 min at 37°C with stirring. The cell preparations were filtered through cell strainers (pore size, 100 µm; BD Biosciences, Franklin Lakes, New Jersey, USA) and then used for flow cytometric analysis.

For flow cytometric analysis, cells were suspended in 2% (vol/vol) newborn calf serum in PBS and treated with anti-CD16/32 antibody (Tru Stain fcX, BioLegend, San Diego, California, USA) to prevent nonspecific staining. The cells were washed and further stained with the following antibodies: phycoerythrin (PE)–anti-CD31 (BD Biosciences), PE–anti-c-kit (BD Biosciences), PE-Cy7–anti-F4/80 (BioLegend), fluorescein isothiocyanate (FITC)–anti-CD34 (BD Biosciences), FITC–anti-Ly6G (BioLegend), FITC–anti-CD63 (gift from Dr. Kurashima, The University of Tokyo) (Kurashima et al., 2012), allophycocyanin (APC)–anti-CD49f (BioLegend), APC–anti-Fc epsilon receptor 1 (FcϵRI, eBioscience, San Diego, California, USA), APC-Cy7–anti-CD11b (BioLegend), and brilliant violet (BV) 421–anti-CD45 (BioLegend). Dead cells were detected by using 7-aminoactinomycin D (7-AAD, BioLegend) and were excluded from analysis. Samples were analyzed MACSQuant (Miltenyi Biotec, Bergish Gladbach, Germany) or FACSAria (BD Biosciences). Cells were isolated using FACSAria through flow cytometry. Keratinocytes are gated as 7-AAD^−^ CD45^−^ CD31^−^ CD34^−^ CD49f^+^ (Saika et al., 2021), and macrophages are gated as 7-AAD^−^ CD45^+^ Ly6G^−^ F4/80^+^ CD11b^+^ cells (Nagatake et al., 2022). These cells are used for RNA extraction to assess gene expression levels. Data analysis was conducted using FlowJo 9.9 software (Tree Star, Ashland, Oregon, USA).

### Vascular permeability assay

The assay was performed as described previously with modifications (Saika et al., 2021). Briefly, at 60 min before euthanasia on day 7 of contact hypersensitivity induction, mice were injected intravenously with 1% (wt/vol) Evans blue dye in PBS. Harvested ears were incubated in 1 M phosphoric acid (Nacalai Tesque) at 37°C overnight to extract the dye. Potassium hydroxide and acetone were added to the extract, and the resultant solution was left to phase-separate at room temperature for at least 30 min. The absorbance (OD_620_) of the aqueous phase was measured in a spectrophotometer (SmartSpec Plus, Bio-Rad Laboratories, Hercules, California, USA).

### Histologic analysis

Analysis was performed as described previously (Saika et al., 2020). Briefly, ear samples were embedded in Tissue-Tek OCT compound (Sakura Finetek, Osaka, Japan), frozen in liquid nitrogen and cut into sections (7 µm) by using a cryostat (CM3050 S, Leica, Wetzlar, Germany). The sections were washed with running water for 10 min, stained with Mayer hematoxylin solution (Wako) for 10 min, and washed with running water for 30 min. The sections were then stained with 1% eosin Y solution (Wako) for 1 min, washed with running water for 10 s, and dehydrated through increasing concentrations of ethanol (1 min at each concentration, 70% to 100%, Nacalai Tesque) and finally in xylene (Nacalai Tesque) for 3 min. They were mounted (Permount, Falma, Tokyo, Japan) and examined under a microscope (BZ-9000, Keyence, Osaka, Japan).

### Isolation and preparation of mast cells

Peritoneal mast cells (PMCs) were prepared as previously reported (Meurer et al., 2016; Sawane et al., 2019). In brief, 9 mL of PBS was injected intraperitoneally into a naïve mouse by using a 20-gauge needle, the abdomen was gently massaged for 1 min to detach peritoneal cells, and then the peritoneal fluid was collected and centrifuged at 400 × *g* and 4°C for 5 min. The pellet was washed with RMPI 1640 medium containing 20% (vol/vol) fetal bovine serum, 100 U/mL penicillin, and 100 µg/mL streptomycin and transferred to a 10-cm dish. Cells were cultured in RMPI 1640 supplemented with 10 ng/mL IL-3 (PeproTech, Cranbury, New Jersey, USA) and 30 ng/mL stem cell factor (PeproTech) in an incubator (37°C and 5% CO_2_) for 2 days, after which the supernatant and non-adherent cells were removed, and fresh culture medium was added. On day 9, the cells were collected by washing the plate three times with PBS (10 mL each time); the cell-containing washes were pooled in a 50-mL tube, which was centrifuged at 400 × *g* for 5 min. The pellet was recovered and moved to a fresh 10-cm dish containing RPMI 1640 supplemented with 20% fetal bovine serum, 100 U/mL penicillin, 100 µg/mL streptomycin, 10 ng/mL IL-3, and 30 ng/mL stem cell factor in an incubator (37°C and 5% CO_2_) for 4 to 5 days. The efficacy of cell recovery and percentage of differentiation to PMCs were assessed by flow cytometry as the FcϵRI^+^ c-Kit^+^ CD45^+^ population; PMC populations that were more than 90% pure were used for the degranulation assay.

### Mast cell degranulation assays

The assays were performed as described previously (Sawane et al., 2019) with modifications. For the IgE-dependent degranulation assay, PMCs were seeded into 96-well plates at 2 × 10^5^ cells/well, incubated for 24 h, and then sensitized with 0.2 mg/mL anti-dinitrophenyl (DNP)–IgE (Sigma-Aldrich, St. Louis, Missouri, USA) for 24 h. Cells were washed twice with Hanks’ Balanced Salt Solution (Nacalai Tesque) and stimulated with 100 ng/mL DNP–bovine serum albumin (BSA; LSL, Tokyo, Japan) for 30 min at 37°C. To assess the effect of lipid metabolites on degranulation, t10,c15-18:2 (final concentration, 300 nM) or 0.1% (vol/vol) ethanol in Hanks’ Balanced Salt Solution as a vehicle control was added to cells 30 min before stimulation with DNP–BSA. The sample size for the control group is n = 5, and for the DNP-BSA stimulated group, it is n = 7/group. For the IgE-independent degranulation assay, PMCs were seeded into 96-well plates at 2 × 10^5^ cells/well and incubated for 24 h, after which adenosine-5’-triphosphate disodium salt hydrate (ATP; final concentration, 0.1 nM) or 2,4,6-trinitrobenzene sulfonic acid (TNBS; final concentration, 1 mM) was added for 1 h. To evaluate baseline and confirm PMCs degranulation levels, we established a control group labeled “naïve,” consisting of unstimulated PMCs that did not receive ATP or TNBS treatment. t10,c15-18:2 (final concentration, 300 nM) or 0.1% (vol/vol) ethanol in PBS as a vehicle control was added to cells 30 min before stimulation with ATP or TNBS. The sample size for the non-stimulation group is n = 5/group, and the stimulated group, it is n = 4 to 6/group. Following the stimulation period, PMCs were kept on ice for 30 min, washed with PBS, and stained on ice with anti-CD63 antibody as a marker for degranulation. The degranulation level of PMCs was measured by flow cytometry.

### Reverse transcription and quantitative real-time PCR analysis

The procedures were performed as described previously (Nagatake et al., 2018). Briefly, total RNA was isolated using Sepazol (Nacalai Tesque) from HaCaT cells or cells sorted from day 7 ear tissue, specifically keratinocytes (7-AAD^−^ CD45^−^ CD31^−^ CD34^−^ CD49f^+^) and macrophages (7-AAD^−^ CD45^+^ Ly6G^−^ F4/80^+^ CD11b^+^). RNA samples were incubated with DNase I (Thermo Fisher Scientific, Waltham, Massachusetts, USA) and reverse transcribed into cDNA by using a Super Script VIRO cDNA Synthesis Kit (Thermo Fisher Scientific). Total RNA was extracted from ear tissues by using a Relia Prep RNA Tissue Miniprep System (Promega) and reverse transcribed. Quantitative real-time PCR analysis was performed by using a LightCycler 480 II (Roche, Basel, Switzerland) and FastStart Essential DNA Probes Master (Roche). Primer sequences were: *Vegfa* forward, 5′-caggctgctgtaacgatgaa-3′; *Vegfa* reverse, 5′-gctttggtgaggtttgatcc-3′; *Actb* forward, 5′-aaggccaaccgtgaaaagat-3′; *Actb* reverse, 5′-gtggtacgaccagaggcatac-3′; *VEGFA* forward, 5’-tgtgtgtgtgtgagtggttga-3’; *VEGFA* reverse, 5’-tctctgtgcctcgggaag-3’; *ACTB* forward, 5’-catgtacgttgctatccaggc-3’; and *ACTB* reverse, 5’-ctccttaatgtcacgcacgat-3’.

### Enzyme-linked immunosorbent assay (ELISA) for vascular endothelial growth factor A

The amount of VEGF-A protein in ear homogenates was analyzed by using a Mouse VEGF Quantikine ELISA Kit (R&D Systems, Minneapolis, Minnesota, USA) according to the manufacturer’s protocol. In brief, ear skin samples were homogenized for 30 s with one 4.8-φ bead and three 3.2-φ beads in PBS containing protease-inhibitor cocktail (Sigma-Aldrich) and centrifuged (9100 × *g*, 20 min, 4°C) as followed previous study (Nagatake et al., 2022). The supernatant was collected and diluted to a protein concentration of 4 mg/mL with PBS containing a protease inhibitor cocktail for ELISA analysis. A microplate reader (Bio-Rad Laboratories) was used to measure absorbance at OD_450_.

### Reporter assays

Fatty acids were tested for their ability to activate nuclear receptors by using human RXRα, RXRβ, and RXRγ luciferase reporter assay systems (Indigo Biosciences, State College, Pennsylvania, USA) according to the manufacturer’s procedure. In brief, reporter cells expressing a hybrid receptor composed of the Gal4 DNA-binding domain fused to the ligand-binding domain of the specific nuclear receptor, together with the firefly luciferase reporter gene, were provided with the reporter assay systems. Reporter cells were incubated with the test compounds (final concentration, 30 µM) for 24 h at 37°C in 5% CO_2_. Light emission was measured in a microplate luminometer (Arvo X2, Perkin Elmer, Waltham, Massachusetts, USA), and the activities of the nuclear receptors were quantified as relative light units.

### HaCaT cell culture

HaCaT cell culture was performed as described previously with some modifications (Saika et al., 2021). HaCaT cells (Boukamp et al., 1988) were obtained from CLS Cell Lines Service (Eppelheim, Germany) and grown in Dulbecco’s modified Eagle’s medium with high glucose (DMEM; Sigma-Aldrich) supplemented with 10% (vol/vol) FBS (Gibco), and 100 U/mL penicillin and 100 µg/mL streptomycin at 37°C and 5% CO_2_. HaCaT cells were seeded in 96-well plates at 3 × 10^4^ cells/well, and cultured for 24 h. Then, the medium was replaced with DMEM without FBS and the cells were treated first with 300 nM t10,c15-18:2 for 30 min and then with 100 ng/mL recombinant human IFN-γ (PeproTech) for 24 h. We used 0.2% (vol/vol) ethanol in DMEM as vehicle control.

### Sample preparation for liquid chromatography–tandem mass spectrometry analysis

Lipids were extracted as previously reported (Nagatake et al., 2022). In brief, for murine serum samples (n = 6 mice/group), 50 µL of serum was added to 450 µL of methanol (Wako Pure Chemicals) and vortexed twice for 10 s each time. For fecal samples (n = 6 mice/group), fecal pellets were combined with five 5-mm zirconia beads (M&S Instruments, Osaka, Japan) in methanol and then homogenized at 6500 rpm by using the Precellys lysis and homogenization system (Bertin Instruments, Montigny-le-Bretonneux, France) twice for 15 s each time. Samples were stored overnight at –30°C for extraction. Samples were centrifuged at 1600 × *g*, 4°C for 10 min. The supernatant (200 µL) was mixed with a deuterium-labeled internal standard (15(*S*)-hydroxyeicosatetraenoic acid-d_8_, Cayman Chemical) and 200 µL water (Wako Pure Chemicals) and centrifuged at 10,000 × *g*, 4°C for 1 min. The supernatant underwent solid-phase extraction using Sep-Pak C_18_ cartridges (Waters, Milford, Massachusetts, USA).

### LC-MS/MS analysis

LC-MS/MS was performed as reported previously (Nagatake et al., 2022). Briefly, lipids were obtained using a Monospin C_18_-AX centrifugal column with deuterium-labeled internal standard. Fatty acid metabolites were analyzed with a Shimadzu LCMS-8050 system with a triple-quadrupole mass spectrometer (Shimadzu, Kyoto, Japan). The chromatographic separation used a Chiralcel OJ-3R column (150 × 4.6 mm, 3.0 μm; Daicel, Osaka, Japan). Solvent A was 0.1% acetic acid, solvent B was methanol, the flow rate was 0.4 mL/min, and the oven temperature was 40 °C. The metabolites were eluted with the following gradient: 10%–75% solvent B from 0−5 min, 75% solvent B for 15 min, 75%–90% solvent B from 20−25 min, 90% solvent B for 10 min, 90%–100% solvent B from 35−50 min, and 100% solvent B for 8 min, with 100%–10% solvent B for 58–59.1 min and 10% solvent B for 0.9 min for column wash and equilibration, respectively. The injection volume was 1 μL. For MS, nitrogen was used as drying gas (flow rate 10 L/min), nebulizing gas (2.5 L/min), and heating gas (10 L/min). The temperatures were set at 400°C for the heat block, 270°C for the ESI interface, and 477°C for the desolvation line. For lipidomic analysis, LC-MS raw data were preprocessed by using LabSolutions (Shimadzu) for peak alignment, noise filtering, and data extraction. Fatty acid levels were normalized as the peak area ratios of each fatty acid to the respective internal standard. Deuterated internal standards were measured to check recoveries of fatty acid metabolites. For the quantification of fatty acid metabolites, calibration curves were drawn by using fatty acid standards.

### Statistical analysis

Data were analyzed by using the non-parametric Kruskal–Wallis test followed by the Dunn multiple-comparison test or the Mann–Whitney U test (Prism 6, GraphPad Software, San Diego, California, USA). A *P* value of less than 0.05 was considered significant.

## Results

### t10,c15-18:2 and c9,c15-18:2 were produced from dietary omega-3 fatty acids through bacteria-dependent metabolism

**Figure 2:**
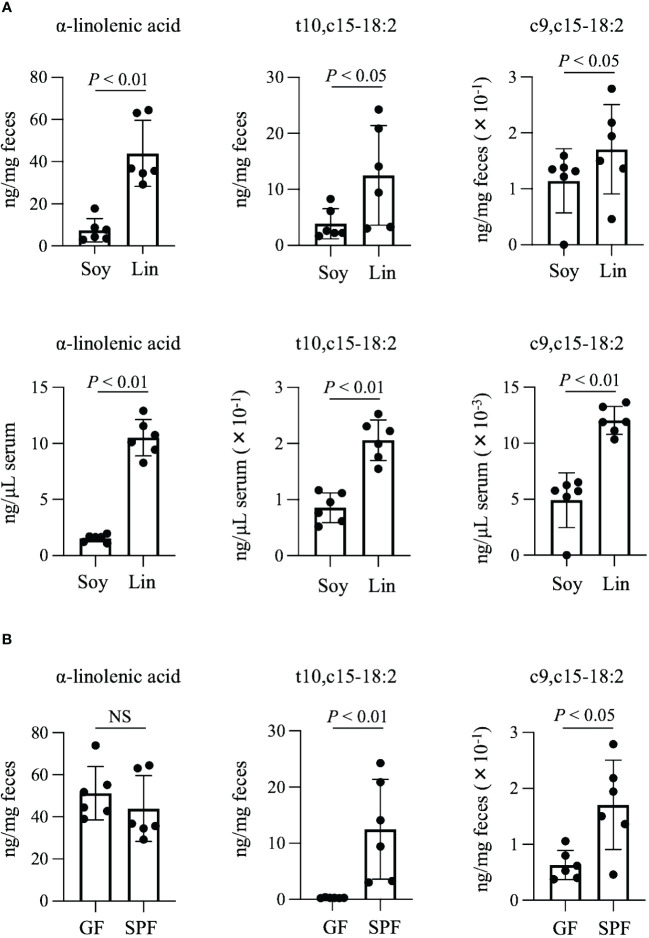
Production of t10,c15-18:2 and c9,c15-18:2 from dietary omega-3 fatty acids. **(A, B)** The concentrations of the fatty acids **(A)** in the feces and serum of SPF mice on a diet containing either soybean oil (Soy) or linseed oil (Lin) and **(B)** in the feces of GF and SPF mice on a Lin-containing diet. The concentrations of the fatty acids were determined by using LC-MS/MS. Each point represents data from an individual mouse (n = 6 mice/group). Statistical significance was evaluated by using the Mann–Whitney U test. NS, not significant.

The fatty acids t10,c15-18:2 and c9,c15-18:2 are end products of bacterial α-linolenic acid saturation metabolism (**Figure 1**). Because increased intake of α-linolenic acid from the diet enhances the production of its derived metabolites (Nagatake et al., 2022), we initially investigated whether dietary omega-3 fatty acid intake increases the levels of t10,c15-18:2 and c9,c15-18:2. We provided mice with diets based either on linseed oil, which is high in omega-3 α-linolenic acid, or on conventional soybean oil, which is rich in omega-6 linoleic acid, and found that the levels of α-linolenic acid, t10,c15-18:2, and c9,c15-18:2 were higher in both the feces and serum from mice on the linseed oil–based diet than from mice on the soybean oil–based diet (**Figure 2A**). To assess whether the presence of these metabolites in mice relied on intestinal bacteria, we administered a linseed oil–based diet to both SPF and GF mice for 2 months. The fecal levels of α-linolenic acid were similar between GF and SPF mice, but the levels of t10,c15-18:2 and c9,c15-18:2 were higher in SPF mice than in GF mice, thus suggesting that the production of these metabolites depends on intestinal bacteria (**Figure 2B**).

### Contact hypersensitivity was ameliorated by treatment with t10,c15-18:2 but not c9,c15-18:2

**Figure 3:**
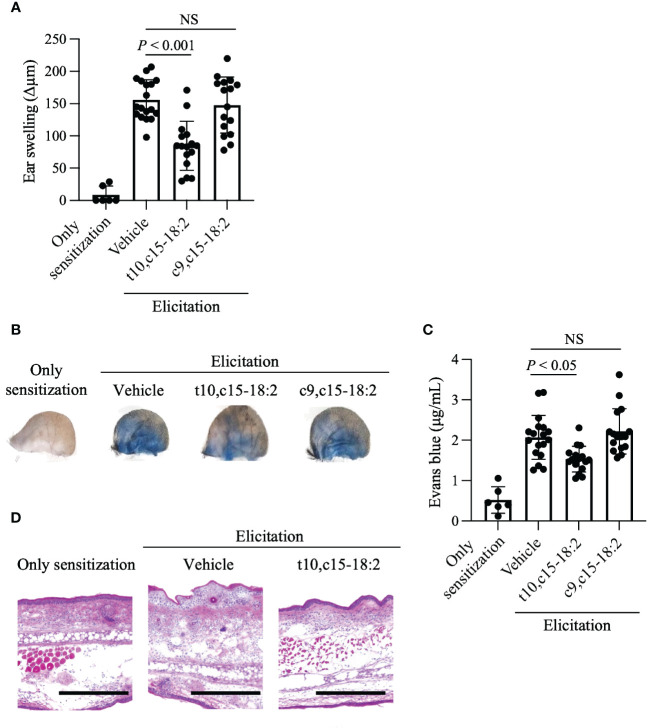
Reduction of ear skin swelling caused by contact hypersensitivity through t10,c15-18:2 treatment. Mice were treated topically with either t10,c15-18:2 or c9,c15-18:2 (dose, 1 μg/mouse) in 50% (vol/vol) ethanol in PBS or the vehicle as a control. **(A)** DNFB-induced ear swelling was evaluated on day 7. The sample sizes for each group are as follows: non-elicitation group, n = 3 mice/group; vehicle-treated group, n = 9 mice/group; t10,c15-18:2-treated group and c9,c15-18:2-treated group, n = 7 mice/group. The data presented in this analysis are the result of combining data from three independent experiments. **(B, C)** Evans blue solution was administered intravenously 60 min before analysis on day 7. **(B)** Representative images of ears. **(C)** Evans blue dye was extracted from ear tissues and quantified via measurement of absorbance at OD_620_. These data are compiled from three independent experiments. The sample sizes for each group are as follows: non-elicitation group, n = 3 mice/group; vehicle-treated group, n = 9 mice/group; t10,c15-18:2-treated group and c9,c15-18:2-treated group, n = 7 mice/group. The data presented in this analysis are the result of combining data from three independent experiments. **(D)** Ear tissue samples procured on day 7 were stained with hematoxylin and eosin for histologic examination. Representative images from two independent experiments are shown. Scale bars represent 100 μm. Statistical significance was evaluated by using the Kruskal–Wallis test followed by Dunn’s multiple-comparison test. NS, not significant.

We then used the mouse model of DNFB-induced allergic contact hypersensitivity to explore the immunomodulatory roles of t10,c15-18:2 and c9,c15-18:2 in this process. Pre-treatment with t10,c15-18:2 reduced ear swelling, a marker of an inflammatory condition in this model, whereas c9,c15-18:2 did not exert a similar effect (**Figure 3A**). Enhancement of vascular permeability plays a pivotal role in the development of ear swelling (Zhang et al., 2006; Yuan et al., 2010; Huggenberger and Detmar, 2011; Ono et al., 2017). To assess vascular leakage at the inflammation site, we performed an experiment involving Evans blue dye. In the DNFB-induced inflamed ears of mice treated with the vehicle only, blue dye was distributed broadly due to vascular leakage after the injection of Evans blue (**Figure 3B**). However, dye distribution was curtailed in the ears of mice treated with t10,c15-18:2, suggesting reduced leakage, whereas it remained widespread despite treatment with c9,c15-18:2 (**Figure 3B**). Quantification of Evans blue extracted from the ear tissue indicated that dye accumulation was reduced after pre-treatment with t10,c15-18:2, but c9,c15-18:2 pre-treatment did not exert this effect (**Figure 3C**). Histologic analysis revealed that—unlike c9,c15-18:2—t10,c15-18:2 inhibited the formation of epidermal edema (spongiosis), a characteristic feature of contact hypersensitivity (**Figure 3D**). These findings indicate that t10,c15-18:2 mitigated skin inflammation in mice, particularly by attenuating vascular permeability.

### t10,c15-18:2 inhibited vascular permeability by reducing VEGF-A production

**Figure 4:**
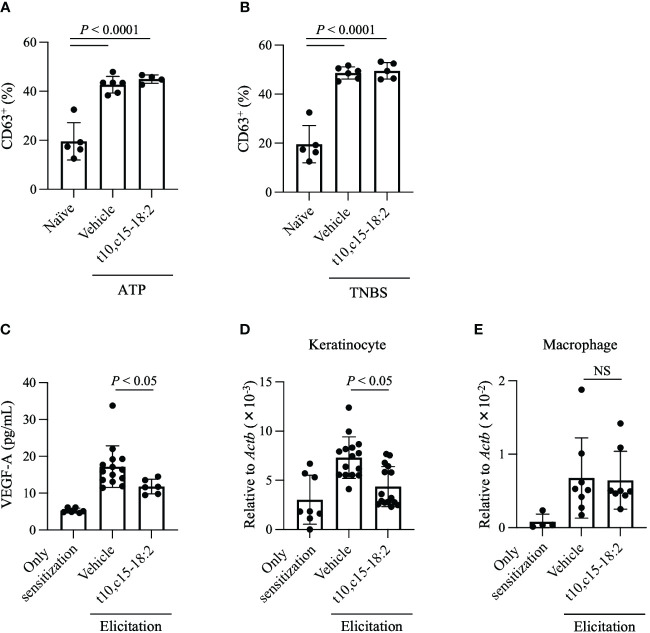
t10,c15-18:2 reduces VEGF-A production and its gene expression in keratinocytes. **(A, B)** The mast cell degranulation assay. PMCs were stimulated by incubation with **(A)** 0.1 nM ATP or **(B)** 1 mM TNBS for 1 h; t10,c15-18:2 (final concentration, 300 nM) or the vehicle (0.1% [v/v] ethanol in PBS) as a control was added 30 min before stimulation. A naïve group consisting of unstimulated PMCs, which did not receive either ATP or TNBS treatment, was also prepared. The degranulation level was measured by flow cytometry using staining for the degranulation marker CD63. For the non-stimulation group, the sample size is n = 5/group, while for the stimulated group, the sample size is n = 4 to 6/group. The data presented here are the result of combining data from two independent experiments. **(C–E)** Mice were treated topically with either t10,c15-18:2 or c9,c15-18:2 (1 μg/mouse) in 50% (vol/vol) ethanol in PBS or the vehicle as a control. **(C)** Ear homogenates were prepared on day 7 and examined by ELISA to determine the amount of VEGF-A. For the non-elicitation group and the fatty acid-treated group, the sample size is n = 6 group, while for the vehicle-treated group, the sample size is n = 14 group. The data are combined from two independent experiments. **(D)** Keratinocytes (7-AAD^−^ CD45^−^ CD31^−^ CD34^−^ CD49f^+^) were sorted from ear tissue on day 7, and quantitative real-time PCR analysis was performed to measure the expression levels of *Vegfa*, which were normalized to those of *Actb*. For the non-elicitation group, n = 8/group; for the elicitated group, n = 15/group. **(E)** macrophages (7-AAD^−^ CD45^+^ Ly6G^−^ F4/80^+^ CD11b^+^) were sorted from ear tissue on day 7, and quantitative real-time PCR analysis was performed to measure the expression levels of *Vegfa*, which were normalized to those of *Actb*. For the non-elicitation group, n = 4/group; for the elicitated group, n = 8/group. The data are combined from four independent experiments for keratinocytes and from two independent experiments for macrophages. Statistical significance was evaluated by using the Kruskal–Wallis test followed by Dunn’s multiple-comparison test. NS, not significant.

Vascular permeability during contact hypersensitivity is heightened via two primary mechanisms related to mast cell degranulation and VEGF-A production (Kunstfeld et al., 2004; Yamamoto et al., 2007; Hoppe et al., 2020). To evaluate whether t10,c15-18:2 inhibited mast cell degranulation, we measured mast cell expression of CD63, a known marker of mast cell degranulation (Kurashima et al., 2012; Sawane et al., 2019). Mast cells undergo degranulation in an IgE-independent fashion during contact hypersensitivity and release pro-inflammatory mediators, including histamine and proteases (Honda et al., 2013). Because this IgE-independent reaction is induced by ATP or haptens, we stimulated PMCs with ATP or TNBS, a water-soluble hapten, and evaluated subsequent degranulation (Manabe et al., 2017; Hoppe et al., 2020). Pre-treatment with t10,c15-18:2 did not diminish the expression of CD63, which increased after mast cell exposure to either ATP or TNBS (**Figures 4A, B**). We also confirmed that t10,c15-18:2 had scant effect on IgE-dependent degranulation (**Supplementary Figure 1**), which plays a minimal role in the DNFB-induced contact hypersensitivity model in C57BL6J mice (Nagai et al., 2000).

We then focused on VEGF-A, a potent regulator of vascular endothelial cells known for its role in enhancing vascular permeability (Shibuya, 2011; Lee et al., 2021). Whereas the VEGF-A level was elevated in ear tissues treated with the vehicle only, its concentration was lower in ear skin treated with t10,c15-18:2 than in vehicle-treated samples (**Figure 4C**). Both keratinocytes and macrophages secrete VEGF-A during skin inflammation (Johnson and Wilgus, 2014); therefore, we assessed *Vegfa* gene expression in keratinocytes and macrophages isolated from the ear tissues. Topical application with t10,c15-18:2 decreased *Vegfa* expression in keratinocytes (**Figure 4D**) but not macrophages (**Figure 4E**). These results indicate that t10,c15-18:2 suppressed *Vegfa* expression in keratinocytes, subsequently reducing edema. In addition, we confirmed that t10,c15-18:2 inhibits *VEGFA* gene expression in keratinocytes using the human keratinocyte cell line, HaCaT cells (**Supplementary Figure 2**), indicating that t10,c15-18:2 directly affects to keratinocyte function.

Furthermore, it has been reported that mice overexpressing VEGF-A in the epidermis failed to down-regulate inflammation in delayed-type hypersensitivity (Kunstfeld et al., 2004), indicating that VEGF-A is a target for reducing skin inflammation. We confirmed that topically applied with t10,c15-18:2 in post-elicitation, specifically on day 6, also reduced ear swelling (**Supplementary Figure 3**). This supports the effect that t10,c15-18:2 is effective in reducing inflammation and swelling following its onset.

### t10,c15-18:2 reduced skin inflammation via RXRs

**Figure 5:**
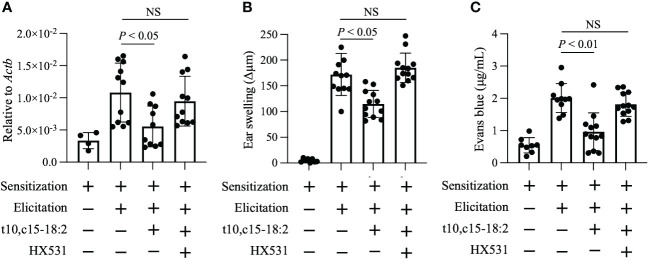
t10,c15-18:2 inhibits skin inflammation in an RXRs-mediated manner. Mice were topically treated with the RXR pan-antagonist HX531 or a vehicle (comprising 50% [vol/vol] dimethyl sulfoxide and 25% [vol/vol] ethanol in PBS) on days 0 and 5 for 60 min and were given either t10,c15-18:2 (1 µg per administration) or a vehicle (50% [vol/vol] ethanol in PBS) for 30 min, followed by DNFB treatment. **(A)** Keratinocytes (7-AAD^−^ CD45^−^ CD31^−^ CD34^−^ CD49f^+^) were sorted from ear skin on day 7, and quantitative real-time PCR analysis was performed to measure the expression levels of *Vegfa*, which were normalized to those of *Actb*. For the non-elicitation group, n = 2 mice/group; for the elicited group, n = 5 to 6 mice/group. **(B)** Ear swelling was evaluated on day 7. For the non-elicitation group, n = 4 mice/group; and for the elicited group, n = 6 mice/group. **(C)** Evans blue dye was extracted from ears and was measured as absorbance at OD_620_. For the non-elicitation group, n = 4 mice/group; for the elicited group, n = 5 to 6 mice/group. The data are combined from two independent experiments. Statistical significance was evaluated by using the Kruskal–Wallis test followed by Dunn’s multiple comparison test. NS, not significant.

RXRs are highly expressed in keratinocytes (Saika et al., 2021); therefore, we explored whether RXRs contribute to the anti-inflammatory action of t10,c15-18:2. We administered HX531, a pan-RXR antagonist, to mice and exposed them to t10,c15-18:2. HX531 administration abolished the inhibitory effect of t10,c15-18:2 on *Vegfa* expression in keratinocytes (**Figure 5A**). Additionally, in contrast to the effects of t10,c15-18:2 alone, co-treatment with t10,c15-18:2 and HX531 failed to reduce ear swelling (**Figure 5B**), a characteristic DNFB-induced symptom of contact hypersensitivity, and enhanced vascular permeability (**Figure 5C**). These results indicate that t10,c15-18:2 attenuated skin inflammation in the mouse model of DNFB-induced contact hypersensitivity through an RXR-dependent pathway, by downregulating vascular permeability via the suppression of *Vegfa* expression in keratinocytes.

**Figure 6:**
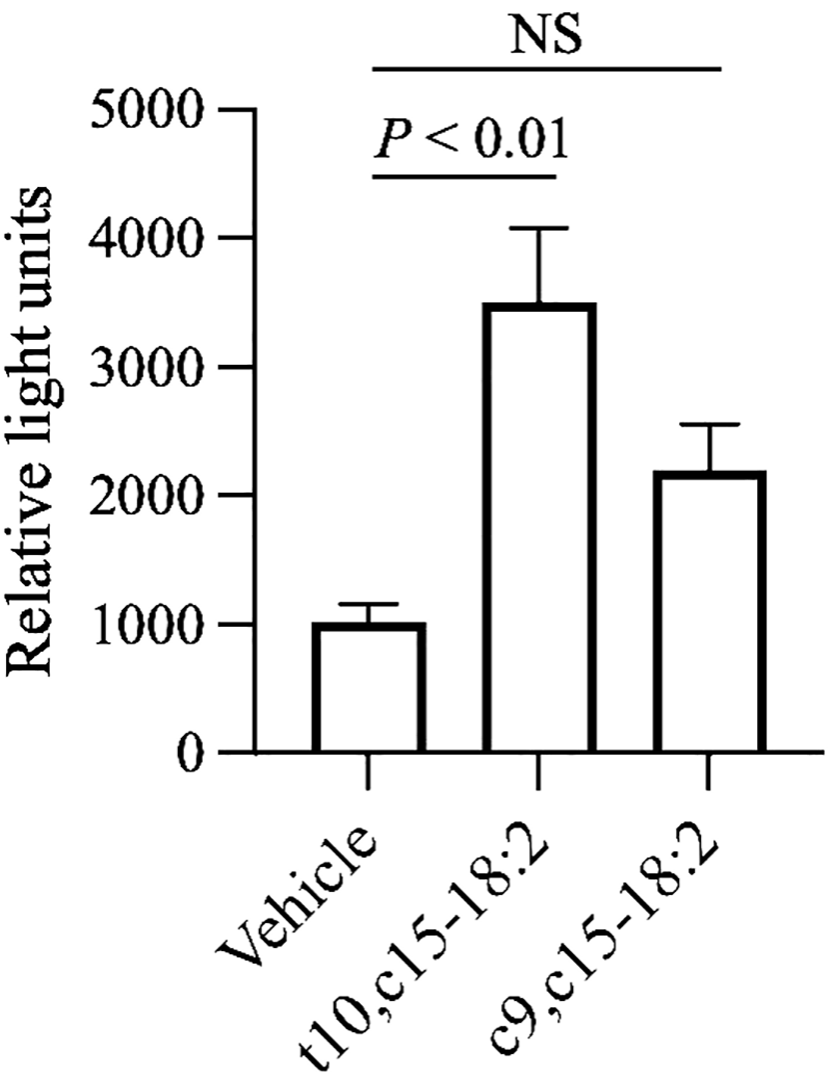
Activation of RXRγ by t10,c15-18:2. The activation level of RXRγ after a 24-h exposure to a fatty acid with a final concentration of 30 μM (n = 6/group) or the vehicle (n = 2/group) was assessed by using a reporter assay system. The data (mean ± SD, *n* = 6) are combined from two independent experiments. Statistical significance was evaluated by using the Kruskal–Wallis test followed by Dunn’s multiple comparison test. NS, not significant.

To assess the ligand activities of RXRα, RXRβ, and RXRγ in our mouse model, we used a luciferase reporter assay. Because c9,c15-18:2 failed to significantly decrease skin inflammation, we used it as a non-functional control for comparison with the ligand activity of t10,c15-18:2. Our findings showed that t10,c15-18:2 had unique RXRγ ligand activity, which was superior to that of c9,c15-18:2 (**Figure 6**). In contrast, neither t10,c15-18:2 nor c9,c15-18:2 had ligand activity for RXRα and RXRβ (**Supplementary Figure 4**). These results suggest the involvement of the RXRγ-mediated pathway during t10,c15-18:2-induced suppression of contact hypersensitivity.

## Discussion

Recent advances in metagenomics, metabolomics analyses, and mechanistic studies using animal models have elucidated the beneficial roles of intestinal bacteria in relation to host diseases (Lin and Zhang, 2017; de Vos et al., 2022). Notably, postbiotics—metabolic byproducts derived from food components processed by the intestinal microbiota—are emerging as potential tools for promoting health. Contemporary research highlights the functions of dietary fatty acids as substrates for the production of postbiotics through bacterial metabolism.

In a previous study, we identified 10-hydroxy-*cis*-12-*cis*-15-octadecadienoic acid (αHYA) and αKetoA as specific intermediate metabolites of α-linolenic acid saturation metabolism by intestinal bacteria (Nagatake et al., 2022). Our current study revealed higher serum and fecal levels of t10,c15-18:2 and c9,c15-18:2 than of their precursor metabolites, αHYA and αKetoA. Both studies used the same linseed oil–based diet and feeding duration. Our results therefore imply that t10,c15-18:2 and c9,c15-18:2, as end-products of α-linolenic acid saturation metabolism, distribute more extensively in the murine body than do the intermediate metabolites αHYA and αKetoA. In addition, our data indicate greater quantities of t10,c15-18:2 in the feces and serum than of c9,c15-18:2. Given that both t10,c15-18:2 and c9,c15-18:2 originate from the same precursor fatty acid, αKetoB (Kishino et al., 2013), our findings suggest that t10,c15-18:2 may either be less susceptible to degradation than is c9,c15-18:2 or that t10,c15-18:2 is produced more efficiently from αKetoB. For instance, studies have indicated that *trans*-unsaturated fatty acids are less susceptible to oxidation than *cis*-unsaturated fatty acids (Sargis and Subbaiah, 2003).

The conversion of αKetoB into t10,c15-18:2 and c9,c15-18:2 is biased depending on environmental conditions (Kishino et al., 2003). The process of bacteria-facilitated fatty acid conversion is influenced by various enzyme-associated factors, including expression levels, activity, substrate specificity, and other characteristics, as well as by the composition of the microbiota. The transformation of α-linolenic acid into t10,c15-18:2 and c9,c15-18:2 involves a series of enzymatic processes. For instance, *L. plantarum* converts α-linolenic acid into t10,c15-18:2 and c9,c15-18:2 through various reactions catalyzed by hydratase/dehydratase, dehydrogenase, isomerase, and enone reductase (Kishino et al., 2013). The metabolism of polyunsaturated fatty acids is not confined to these enzymes and encompasses others originating from a variety of bacteria. Numerous bacterial species facilitate the transformation of unsaturated fatty acids into hydroxy fatty acids, including *Bifidobacterium* spp., *Streptococcus* spp., *Clostridium* spp., *Lactobacillus* spp., *Lactiplantibacillus* spp., *Pseudomonas* spp., and *Corynebacterium* spp (Rosberg-Cody et al., 2011; O'Connell et al., 2013; Ogawa et al., 2018). Some of these bacteria harbor proteins known as ‘myosin-cross-reactive antigens,’ which exhibit fatty acid hydratase activity. In addition, *Escherichia coli* and *Pseudomonas aeruginosa* have been identified as having dehydrase activity (Moynie et al., 2013; Chen et al., 2022). These bacterial enzymes also are considered to play a role in the production of t10,c15-18:2 from α-linolenic acid, suggesting that multiple metabolic pathways involving various intestinal bacteria might contribute to the production of t10,c15-18:2. Such a perspective indicates the intricate metabolic interactions within the microbiota. The vast network of pathways leading to the synthesis of specific beneficial postbiotic fatty acids, such as t10,c15-18:2, underscores the importance of gaining a comprehensive understanding of these processes for potential therapeutic applications.

We discovered that t10,c15-18:2 and c9,c15-18:2 have distinctly different effects on skin inflammation. The location and specific placement of double bonds within fatty acids significantly influence the structure, dynamics, and signaling functions of biological membranes as well as their ability to influence physiologic functions (Leger et al., 1990; Perillo et al., 2012). For example, conjugated linoleic acids (CLAs), including c9,t11-CLA and t10,c12-CLA, share several overlapping physiologic functions but exhibit different roles in various diseases (Basak and Duttaroy, 2020). Specifically, c9,t11-CLA has numerous neurobiologic effects, including enhancing the proliferation of neuronal progenitor cells and providing protection from glutamate-induced or neuronal cell death; these effects are less pronounced with t10,c12-CLA (Hunt et al., 2010; Fujita et al., 2021). In addition, c9,t11-CLA exhibits a stronger activity than t10,c12-CLA against various cancer cells (Beppu et al., 2006). In contrast, t10,c12-CLA is more effective than c9,t11-CLA in reducing obesity (Miller et al., 2008). These functional variations are considered to stem from differences in receptor-ligand activation potencies. For example, c9,t11-CLA displays higher ligand activity for PPARγ than t10,c12-CLA, serving as a potent agonist, whereas t10,c12-CLA acts as an antagonist for PPARγ, competing with the ligand (Miller et al., 2008). While it is important to consider the potential variations in uptake activity into the cytoplasm or tissue based on the fatty acid structure, these functional differences are considered to arise from variations in receptor-ligand activation potencies. The activation potencies of c9,t11-CLA and t10,c12-CLA differ in regard to PPARα and PPARβ activity (Moya-Camarena et al., 1999; Clement et al., 2002; Granlund et al., 2003). These observations indicate that receptor activation level is modulated by the specific positioning of double bonds within fatty acids, suggesting that the different activities of t10,c15-18:2 and c9,c15-18:2 in terms of their anti-inflammatory activity may be attributed to their respective ligand activities. Our results from the luciferase reporter assay demonstrated that t10,c15-18:2 acts as an RXRγ ligand, and further inhibitor studies indicate that it might be a functional receptor. However, it is important to note that the functions of RXRγ have not been studied as extensively as those of the more widely recognized RXRα and RXRβ (Nunez et al., 2010; Pekow and Bissonnette, 2014; Watanabe and Kakuta, 2018; Zeng et al., 2022).

*VEGFA* expression is upregulated not only in allergic contact dermatitis but also in other inflammatory skin conditions, including atopic dermatitis and psoriasis (Bhushan et al., 1999; Bae et al., 2010; Samochocki et al., 2016). Indeed, transgenic mice that overproduce VEGF-A exhibit exacerbated inflammation, with a self-amplifying loop of fluid leakage and inflammation in the skin, leading to increased fluid accumulation (Xia et al., 2003; Kunstfeld et al., 2004). In the context of inflammation, *VEGF* expression is modulated by a multitude of regulatory mechanisms, including transcription factors and various other stimuli such as growth factors, hormones, cytokines, and cellular stress, such that thoroughly comprehending the regulatory mechanisms of VEGF is a complex task (Arcondeguy et al., 2013). The transcription factor Sp1 has been identified as a pivotal modulator of *VEGF* expression, whereas Sp3 represses Sp1-mediated transcription (Pages and Pouyssegur, 2005). The balance between Sp1 and Sp3 shows the intricate dynamic within the transcriptional regulation of *VEGF* (Hagen et al., 1994). The interaction between Sp1 and Sp3 raises the intriguing possibility that t10,c15-18:2 could stimulate Sp3 via RXRγ, consequently downregulating VEGF expression at the transcriptional level. In another possibility, the murine VEGF promoter is regulated by the concerted action of hypoxia-induced transcription factors such as hypoxia-inducible factor (HIF) and nuclear factor-κ B (NF-κB), which are important for optimal *VEGF* expression (Lukiw et al., 2003; Schmidt et al., 2007). Notably, JunB deficiency leads to reduced HIF and NF-κB–induced *VEGF* expression (Schmidt et al., 2007). We wonder whether t10,c15-18:2 treatment might suppress JunB expression, subsequently diminishing VEGF expression. Our findings indicate the potential to develop innovative treatments to decrease inflammation in allergic contact dermatitis by modulating *VEGFA* expression through RXRγ-mediated signalling. Moreover, targeting the RXRγ–VEGF axis may offer several therapeutic advantages, particularly in alleviating vascular permeability. To harness these opportunities effectively, a deeper comprehension of RXRγ’s role in skin inflammation is imperative and a more comprehensive investigation into the RXRγ–VEGF axis is needed for the advancement of therapies for skin diseases. In this context, future research should delve into the specific molecular mechanisms responsible for the downregulation of *VEGF* expression.

Our findings indicate the potential of t10,c15-18:2 in the development of an effective therapeutic to regulate vascular permeability in allergic contact dermatitis. Notably, with its structural simplicity and lack of conjugated double bonds, t10,c15-18:2 may be less prone to oxidation than CLAs (Zhang and Chen, 1997), supporting the potential of t10,c15-18:2 as a valuable health-promoting compound. In the context of postbiotics, the provision of essential substrates, such as omega-3 fatty acids, along with a selection of probiotics rich in metabolic enzymes for the conversion of these substrates to t10,c15-18:2, may enhance the production of these beneficial postbiotics, thereby bestowing additional health benefits. At the same time, it is important to recognize the limitations of our research. This study was conducted solely with female mice due to their lower aggression levels, which reduces physical skin irritations from behaviors like scratching and mounting (Schwarz et al., 2023). This decision, while beneficial for controlling experimental variables, limits the applicability of our findings across sexes. Therefore, future research should include both male and female mice to comprehensively evaluate sex as a biological variable in allergic contact dermatitis responses. Also, given the nature of t10,c15-18:2, its application to the skin rather than oral administration seems to be the best route to obtain anti-inflammatory effects. Treatment centering primarily on VEGF-centric approaches—anti-VEGF therapy—effectively diminishes inflammation in various conditions, including chronic inflammatory diseases, diabetic macular edema, psoriasis-related skin inflammation, and allergic contact dermatitis (Ardelean et al., 2014; Apte et al., 2019; Luengas-Martinez et al., 2020; Imazeki et al., 2021). At present, therapies targeting VEGF-A are used in treating, for example, age-related macular degeneration and cancer (Ferrara et al., 2007; Ferrara and Adamis, 2016). Given our insights into the activity of t10,c15-18:2 through topical application, the potential exists for its use in alleviating allergic contact hypersensitivity and other conditions marked by increased vascular permeability via VEGF modulation.

## Data availability statement

The original contributions presented in the study are included in the article/**Supplementary Material**. Further inquiries can be directed to the corresponding author.

## Ethics statement

The animal study was approved by Committee on the Ethics of Animal Experiments at NIBIOHN. The study was conducted in accordance with the local legislation and institutional requirements.

## Author contributions

AS: Writing – original draft, Writing – review & editing, Investigation. TN: Writing – review & editing, Investigation. ShK: Writing – review & editing, Resources. NK: Writing – review & editing, Resources. TH: Writing – review & editing, Methodology. KH: Writing – review & editing. PT: Writing – review & editing, Methodology. EN: Writing – review & editing, Investigation. SoK: Writing – review & editing. SaK: Writing – review & editing. KI: Writing – review & editing. KK: Writing – review & editing, Methodology. JO: Writing – review & editing, Resources. JK: Writing – original draft, Writing – review & editing, Supervision.

## Supplementary material

Supplementary Figure 1 Influence of t10,c15-18:2 on IgE-dependent mast cell degranulation. In the mast cell degranulation assay, peritoneal mast cells were sensitized through exposure to 0.2 mg/mL anti-dinitrophenyl (DNP)–IgE for 24 h at 37°C and stimulated with 100 ng/mL DNP–BSA for 30 min at 37°C. To assess the effect of fatty acid metabolites on degranulation, t10,c15-18:2 in 0.1% (vol/vol) ethanol in Hank’s Balanced Salt Solution (final concentration, 300 nM) or the vehicle only was added before the 30-min stimulation with DNP–BSA. For the control group, n = 5/group, and for the DNP-BSA stimulated group, n = 7/group. The degranulation level was measured by using flow cytometry after staining for the degranulation marker CD63. Statistical significance was evaluated by using the Kruskal–Wallis test followed by Dunn’s multiple-comparison test.

Supplementary Figure 2
*VEGFA* gene expression levels in response to t10,c15-18:2 on HaCaT cells. HaCaT cells were obtained from CLS Cell Lines Service (Eppelheim) and cultured in Dulbecco’s modified Eagle’s medium (DMEM) with high glucose. The medium was supplemented with 10% fetal bovine serum, 100 U/mL penicillin, and 100 µg/mL streptomycin. The cells were maintained at 37°C with 5% CO_2_. For the experiments, HaCaT cells were seeded in 96-well plates at a density of 3 × 10^4^ cells/well and cultured for 24 h. Subsequently, the culture medium was replaced with DMEM without fetal bovine serum. The cells were then treated with 300 nM t10,c15-18:2 for 30 min, followed by stimulation with 100 ng/mL recombinant human IFN-γ for 24 h. A vehicle control containing 0.2% (vol/vol) ethanol in DMEM was used for comparison. The data are combined from two independent experiments (n = 11/group).

Supplementary Figure 3 Reduction of the ear skin swelling when t10,c15-18:2 is applied after the elicitation phase. Mice received 0.5% (vol/vol) DNFB on day 0 on the abdominal skin, followed by the challenge of both sides of the ears with 0.2% (vol/vol) DNFB on day 5. After elicitation, mice were topically treated with t10,c15-18:2 (dose, 1 μg/mouse) in 50% (vol/vol) ethanol in PBS, or the vehicle as a control on day 6. DNFB-induced ear swelling was evaluated on day 7. For the non-elicitation group and the vehicle-treated group, n = 4 mice/group; for the t10,c15-18:2-treated group, n = 6 mice/group. The data are combined from two independent experiments.

Supplementary Figure 4 Activation levels of RXRα and RXRβ in response to t10,c15-18:2 and c9,c15-18:2. Activation levels of the nuclear receptors RXRα and RXRβ were assessed by using a reporter assay system after 24-h exposure to fatty acid (final concentration, 30 μM) or the vehicle only. One replicate for each condition.
