# Supplementary figures and images for: The omega-3 postbiotic trans-10-cis-15-octadecadienoic acid attenuates contact hypersensitivity in mice through downregulation of vascular endothelial growth factor A
Source: Front Cell Infect Microbiol. 2024 May 22;14:1355679. doi: 10.3389/fcimb.2024.1355679 (PMC11151274; doi:10.3389/fcimb.2024.1355679)

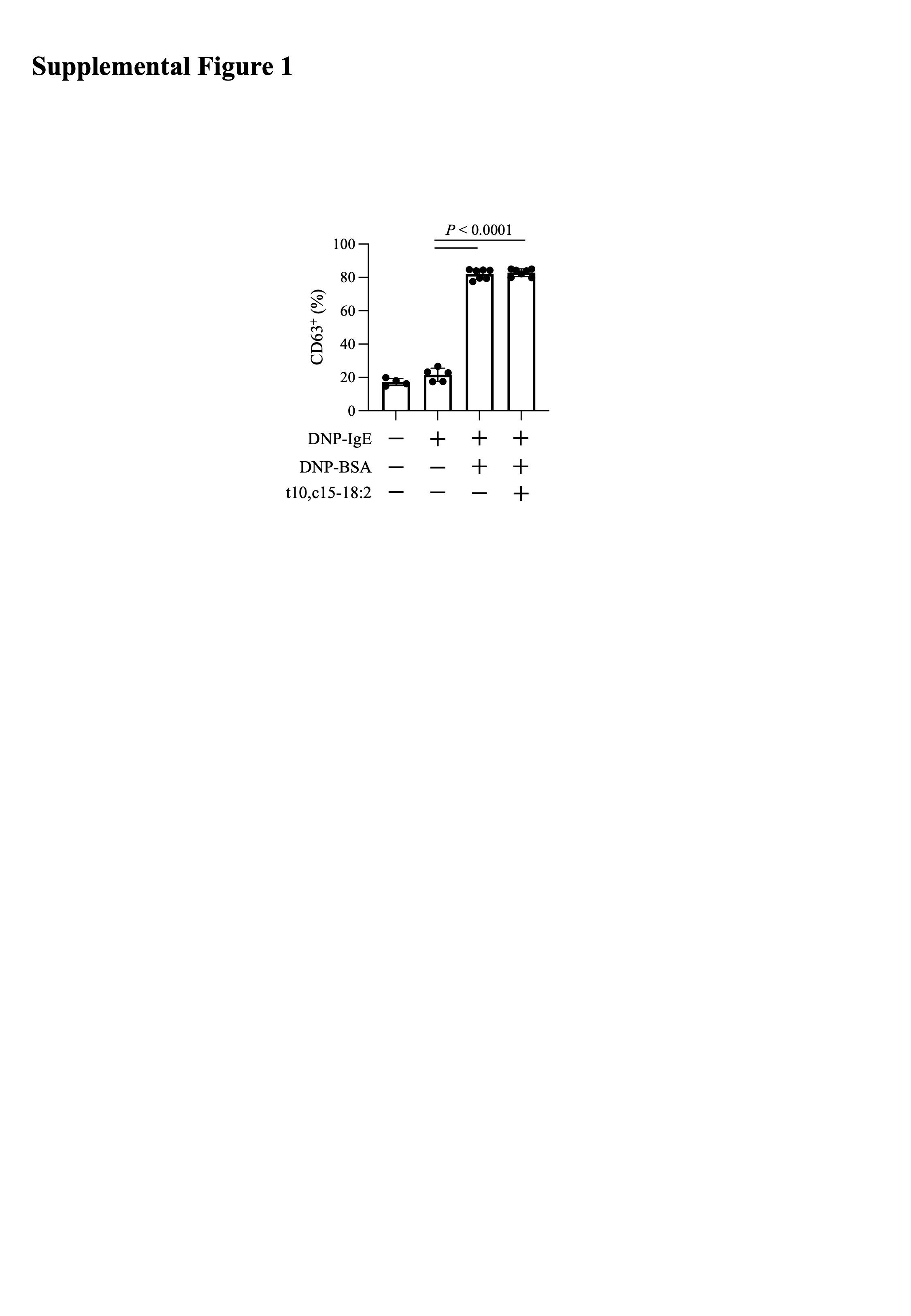

Supplement: Supplementary Figure 1 — Influence of t10,c15-18:2 on IgE-dependent mast cell degranulation. In the mast cell degranulation assay, peritoneal mast cells were sensitized through exposure to 0.2 mg/mL anti-dinitrophenyl (DNP)–IgE for 24 h at 37°C and stimulated with 100 ng/mL DNP–BSA for 30 min at 37°C. To assess the effect of fatty acid metabolites on degranulation, t10,c15-18:2 in 0.1% (vol/vol) ethanol in Hank’s Balanced Salt Solution (final concentration, 300 nM) or the vehicle only was added before the 30-min stimulation with DNP–BSA. For the control group, n = 5/group, and for the DNP-BSA stimulated group, n = 7/group. The degranulation level was measured by using flow cytometry after staining for the degranulation marker CD63. Statistical significance was evaluated by using the Kruskal–Wallis test followed by Dunn’s multiple-comparison test. [file Image_1.jpeg]

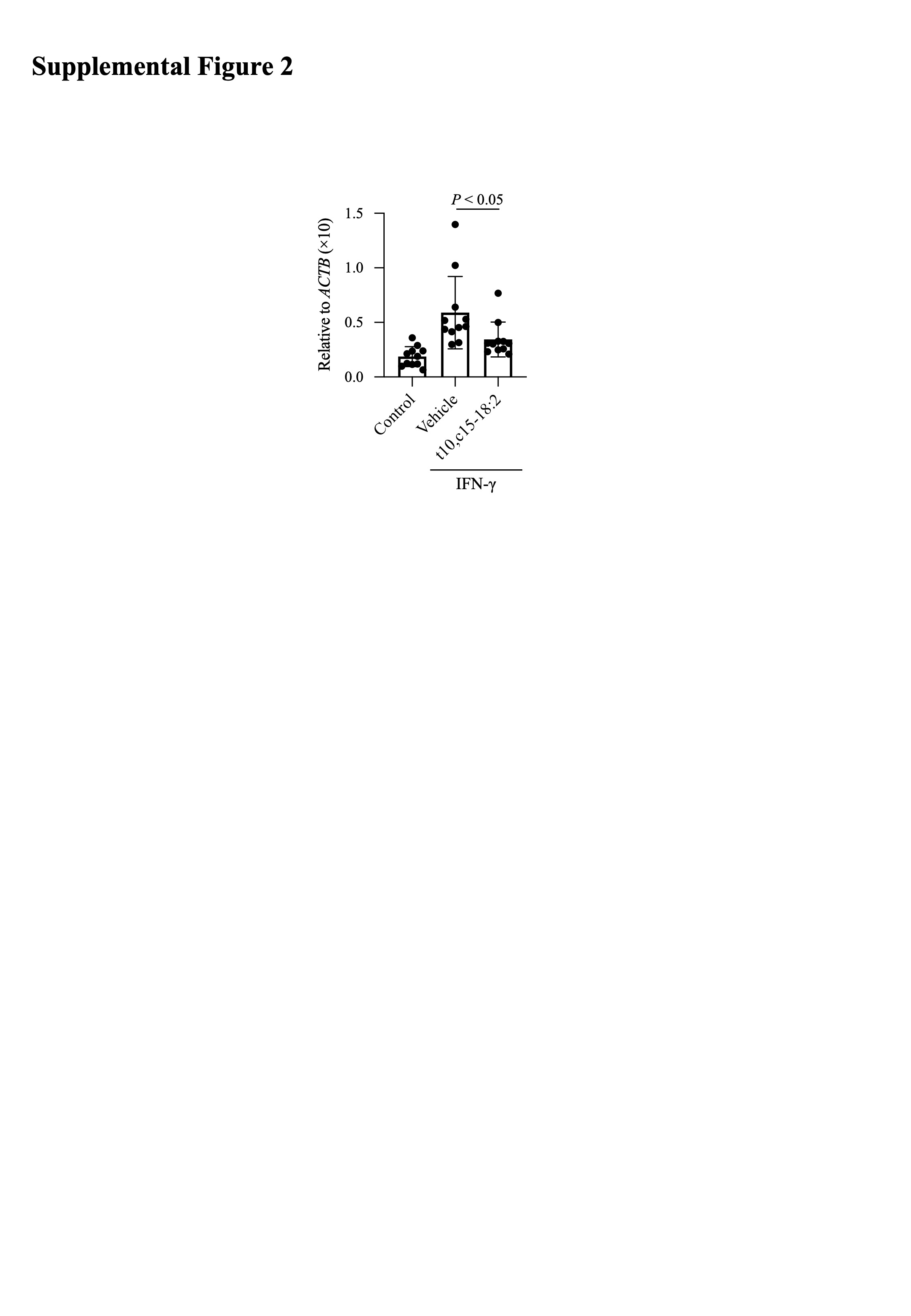

Supplement: Supplementary Figure 2 — VEGFA gene expression levels in response to t10,c15-18:2 on HaCaT cells. HaCaT cells were obtained from CLS Cell Lines Service (Eppelheim) and cultured in Dulbecco’s modified Eagle’s medium (DMEM) with high glucose. The medium was supplemented with 10% fetal bovine serum, 100 U/mL penicillin, and 100 µg/mL streptomycin. The cells were maintained at 37°C with 5% CO2. For the experiments, HaCaT cells were seeded in 96-well plates at a density of 3 × 10^4^ cells/well and cultured for 24 h. Subsequently, the culture medium was replaced with DMEM without fetal bovine serum. The cells were then treated with 300 nM t10,c15-18:2 for 30 min, followed by stimulation with 100 ng/mL recombinant human IFN-γ for 24 h. A vehicle control containing 0.2% (vol/vol) ethanol in DMEM was used for comparison. The data are combined from two independent experiments (n = 11/group). [file Image_2.jpeg]

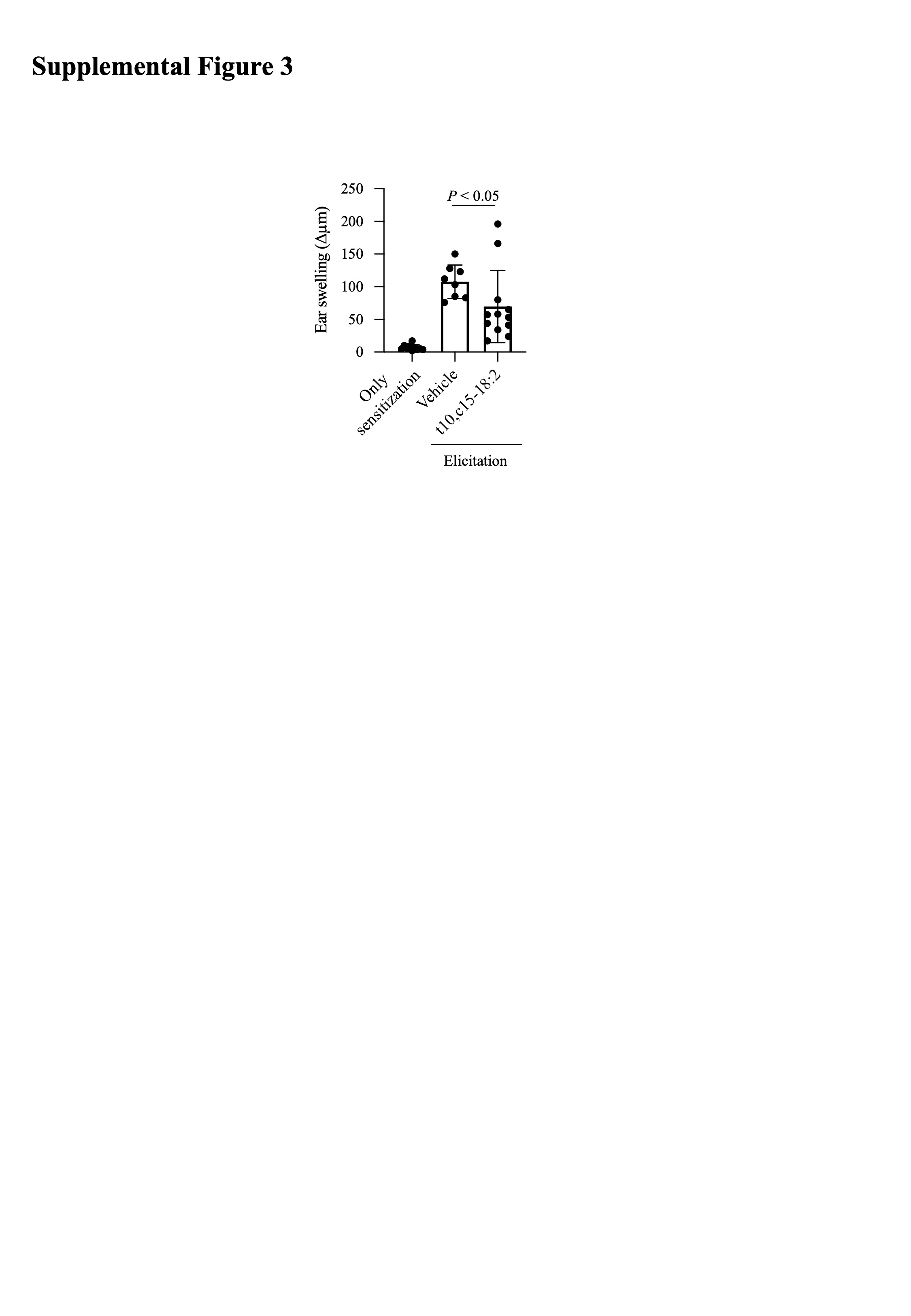

Supplement: Supplementary Figure 3 — Reduction of the ear skin swelling when t10,c15-18:2 is applied after the elicitation phase. Mice received 0.5% (vol/vol) DNFB on day 0 on the abdominal skin, followed by the challenge of both sides of the ears with 0.2% (vol/vol) DNFB on day 5. After elicitation, mice were topically treated with t10,c15-18:2 (dose, 1 μg/mouse) in 50% (vol/vol) ethanol in PBS, or the vehicle as a control on day 6. DNFB-induced ear swelling was evaluated on day 7. For the non-elicitation group and the vehicle-treated group, n = 4 mice/group; for the t10,c15-18:2-treated group, n = 6 mice/group. The data are combined from two independent experiments. [file Image_3.jpeg]

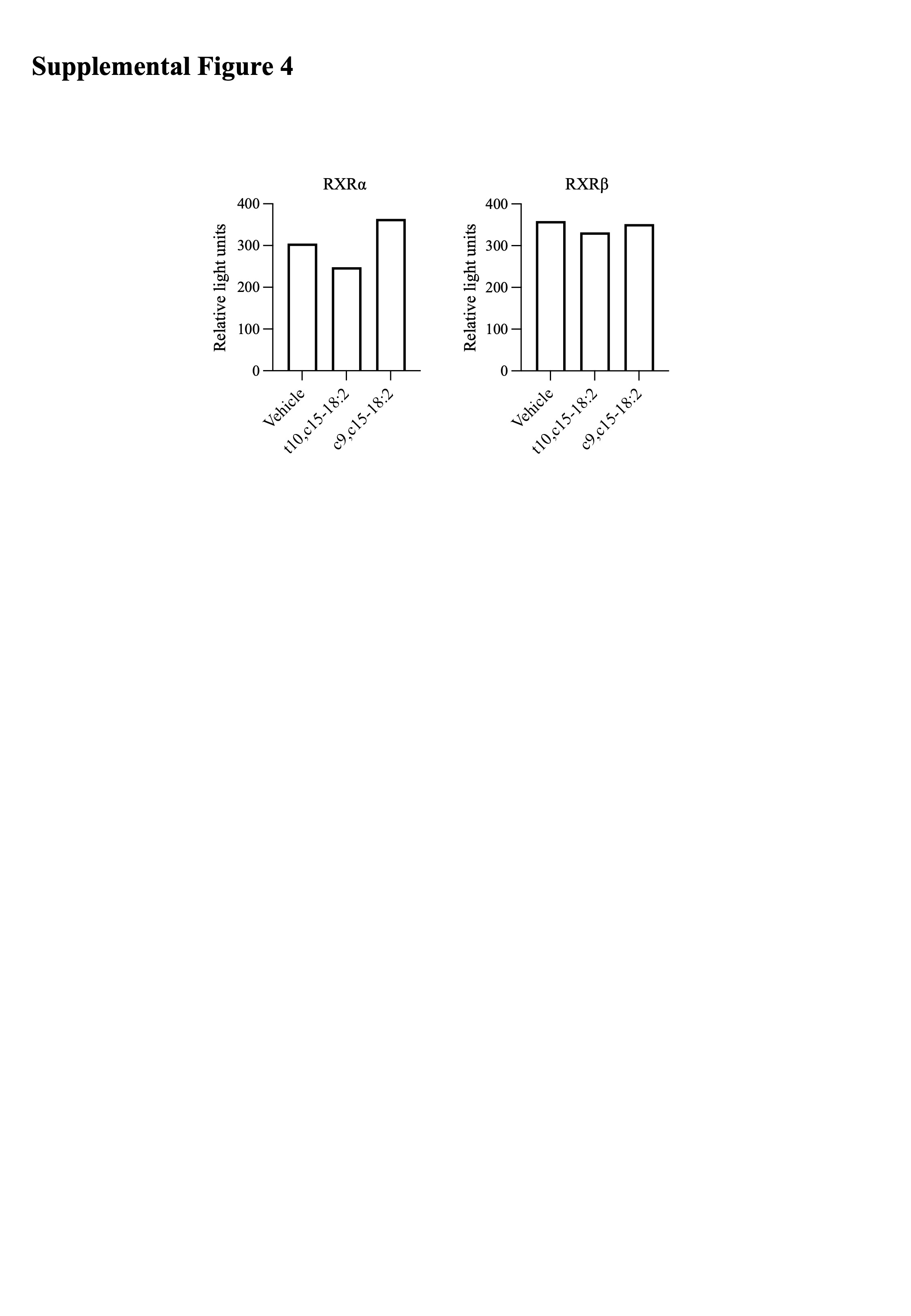

Supplement: Supplementary Figure 4 — Activation levels of RXRα and RXRβ in response to t10,c15-18:2 and c9,c15-18:2. Activation levels of the nuclear receptors RXRα and RXRβ were assessed by using a reporter assay system after 24-h exposure to fatty acid (final concentration, 30 μM) or the vehicle only. One replicate for each condition. [file Image_4.jpeg]
